# Using Psychological Artificial Intelligence (Tess) to Relieve Symptoms of Depression and Anxiety: Randomized Controlled Trial

**DOI:** 10.2196/mental.9782

**Published:** 2018-12-13

**Authors:** Russell Fulmer, Angela Joerin, Breanna Gentile, Lysanne Lakerink, Michiel Rauws

**Affiliations:** 1 Northwestern University Evanston, IL United States; 2 X2AI Inc San Francisco, CA United States; 3 Saxion University of Applied Sciences Enschede Netherlands

**Keywords:** artificial intelligence, mental health services, depression, anxiety, students

## Abstract

**Background:**

Students in need of mental health care face many barriers including cost, location, availability, and stigma. Studies show that computer-assisted therapy and 1 conversational chatbot delivering cognitive behavioral therapy (CBT) offer a less-intensive and more cost-effective alternative for treating depression and anxiety. Although CBT is one of the most effective treatment methods, applying an integrative approach has been linked to equally effective posttreatment improvement. Integrative psychological artificial intelligence (AI) offers a scalable solution as the demand for affordable, convenient, lasting, and secure support grows.

**Objective:**

This study aimed to assess the feasibility and efficacy of using an integrative psychological AI, Tess, to reduce self-identified symptoms of depression and anxiety in college students.

**Methods:**

In this randomized controlled trial, 75 participants were recruited from 15 universities across the United States. All participants completed Web-based surveys, including the Patient Health Questionnaire (PHQ-9), Generalized Anxiety Disorder Scale (GAD-7), and Positive and Negative Affect Scale (PANAS) at baseline and 2 to 4 weeks later (T2). The 2 test groups consisted of 50 participants in total and were randomized to receive unlimited access to Tess for either 2 weeks (n=24) or 4 weeks (n=26). The information-only control group participants (n=24) received an electronic link to the National Institute of Mental Health’s (NIMH) eBook on depression among college students and were only granted access to Tess after completion of the study.

**Results:**

A sample of 74 participants completed this study with 0% attrition from the test group and less than 1% attrition from the control group (1/24). The average age of participants was 22.9 years, with 70% of participants being female (52/74), mostly Asian (37/74, 51%), and white (32/74, 41%). Group 1 received unlimited access to Tess, with daily check-ins for 2 weeks. Group 2 received unlimited access to Tess with biweekly check-ins for 4 weeks. The information-only control group was provided with an electronic link to the NIMH’s eBook. Multivariate analysis of covariance was conducted. We used an alpha level of .05 for all statistical tests. Results revealed a statistically significant difference between the control group and group 1, such that group 1 reported a significant reduction in symptoms of depression as measured by the PHQ-9 (*P*=.03), whereas those in the control group did not. A statistically significant difference was found between the control group and both test groups 1 and 2 for symptoms of anxiety as measured by the GAD-7. Group 1 (*P*=.045) and group 2 (*P*=.02) reported a significant reduction in symptoms of anxiety, whereas the control group did not. A statistically significant difference was found on the PANAS between the control group and group 1 (*P*=.03) and suggests that Tess did impact scores.

**Conclusions:**

This study offers evidence that AI can serve as a cost-effective and accessible therapeutic agent. Although not designed to appropriate the role of a trained therapist, integrative psychological AI emerges as a feasible option for delivering support.

**Trial Registration:**

International Standard Randomized Controlled Trial Number: ISRCTN61214172; https://doi.org/10.1186/ISRCTN61214172.

## Introduction

### Background

Approximately 20 million college students suffer from mental illness in the United States alone [1]. More than 50% of college students report experiencing symptoms of depression and anxiety that impact daily functioning within the last year [2]. Despite a clear need for clinical services, up to 75% of college students do not access adequate mental health care [3]. With a growing desire for on-demand services that engage students and reduce stigma, Web- and mobile-based mental health interventions offer a scalable solution.

Mental health care solutions such as computer-assisted therapy (CAT) have been shown to be a less-intensive and more cost-effective method to deliver empirically validated treatments for depression and anxiety [4,5]. Although traditional in-person treatment remains the standard of care for those with clinical levels of depression, preliminary studies suggest that self-help computer-based cognitive and behavioral interventions produce similar outcomes [6] and are efficacious in the treatment of subthreshold mood disorders.

Moreover, 1 study revealed that nearly 70% of patients expressed interest in using mobile health (mHealth) apps to self-monitor and self-manage their mental health [7]. Early evidence suggests that patients open up more while using an mHealth app than during face-to-face therapy [8]. With 1 app for patients suffering from suicidal thoughts, more subjects reported suicidal ideation using the app than they did on the traditionally administered Patient Health Questionnaire (PHQ-9) [9]. Psychological artificial intelligence (AI) delivering cognitive behavioral therapy (CBT) has been shown to be a feasible, engaging, and effective solution for reducing symptoms of depression and anxiety in college students [10-12]. However, the efficacy of using psychological AI to deliver integrative mental health care, including CBT, requires further exploration. Although CBT is one of the most effective methods for treating anxiety and depression, evidence shows that alternative forms of therapy lead to equally successful outcomes [13]. Applying an integrative approach to therapy for treating patients with depression has been linked to similar levels of posttreatment improvement as those receiving cognitive therapy [14].

### Objective

The objective of this study was to assess the efficacy of using the integrative psychological AI, Tess, to reduce symptoms of depression and anxiety in an engaging way. Tess was designed to deliver personalized conversations based on the expressed emotions and mental health concerns of participants, not to replace trained therapists. Tess focuses on language as the most explicit form of communication, with the proposition that communication between people reveals individual conceptualizations of specific emotions (unpublished data [15]) [16].

This study compared outcomes from 2 to 4 weeks of using integrative psychological AI (Tess) with an information control group (National Institute of Mental Health’s [NIMH] eBook) in a nonclinical college population. It was hypothesized that engaging in conversations with Tess would lead to greater improvement in symptoms relative to the information control group. In addition, we predicted that the duration of time in which participants interacted with Tess would impact the level of symptom reduction. To assess this, participants in the test group were randomly selected to participate in 1 of the 2 groups, which received either 2 or 4 weeks of unlimited access to Tess.

## Methods

The methodology of our study was developed to closely parallel that of Fitzpatrick et al [10] to further contribute to the emergence of AI as a supportive mental health tool. A major goal of our research was to compare findings across conversational agents, in this case, Tess and Woebot. By aligning our research design, participant procedures, and primary outcome measures with the Fitzpatrick study, we intended to assess whether an alternative AI chatbot, Tess, could also yield positive outcomes and effectively reduce symptoms of depression and anxiety. This parallel approach was designed to bolster the broader literature about conversational agents as therapeutic tools. The primary methodological difference was the use of a different AI chatbot rather than the platform evaluated by Fitzpatrick et al [10].

### Recruitment

Participants were recruited using a flyer (Multimedia Appendix 1) distributed to professors and alumni via email and posted through social media outlets such as Facebook and university community channels targeting students across 15 universities across the United States. Inclusion criteria included current enrollment at a university in the United States, aged 18 years and older (screened at the first level via checkbox confirmation), and able to read English (implied). To guard against compromise, for example, from malicious bots, all potential participants were sent an email requesting that they respond using their university email denoting their confirmation.

**Figure 1:**
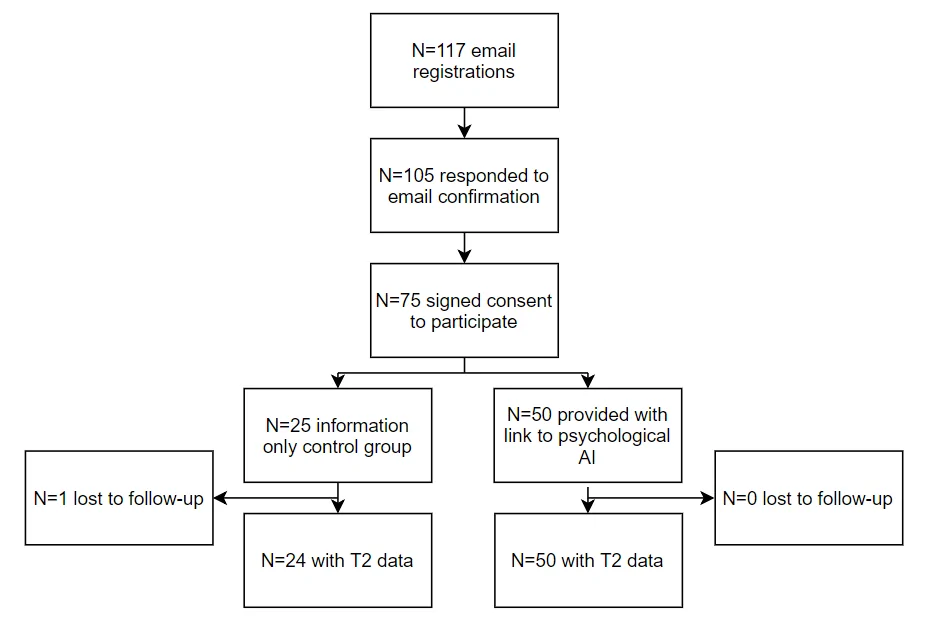
Participant recruitment flow. AI: artificial intelligence.

Confirmed participants were randomized via a computer algorithm that automatically generated a number between 0 and 2 (Figure 1). All participants completed the baseline questionnaires. Participants with number 0 were sent a link to NIMH’s eBook [17] on depression among college students. Participants with numbers 1 and 2 were allocated to receive a direct link to begin chatting with Tess via an instant messenger app. To assess the impact on duration of access on symptom reduction, group 1 participants were granted unlimited access to Tess for 2 weeks and group 2 participants for 4 weeks. Because the randomization allocation occurred algorithmically, allocation concealment was in place. However, the condition to which each participant was allocated was not masked for the service providers (Tess). After approximately 2 weeks (group 1) or 4 weeks (group 0+2), participants were contacted again to complete a second set of questionnaires online. Participants were offered a prorated incentive of US $20 for completion of both assessments.

### Ethics and Informed Consent

This study was retrospectively registered under the trial number of ISRCTN61214172, and involved a nonclinical population of college students. Refer to the Multimedia Appendix 2 for this study’s Consolidated Standards of Reporting Trials-eHealth checklist [18].

Participants indicated their consent to the study’s terms via a checkbox (e-signature) on a closed form (Multimedia Appendix 3) sent via email. The consent informed participants about the study criteria, intervention details, confidentiality, incentives, participant rights, and whom to contact for questions or request to withdraw. Participants who expressed suicidal ideation or self-harm were provided with numbers to a crisis helpline and suicide prevention text line. A total of 75 participants signed the informed consent, and 74 of those completed this study with 0% attrition from the test group and less than 1% attrition from the control group (1/24).

The third author of this paper served as an external representative to support data collection and analysis, which was provided by X2AI Inc. All data, including usage, was deidentified so as to protect the privacy of participants and was reported in aggregate only.

### Interventions

#### Tess

Tess is a psychological AI chatbot designed by X2AI Inc to deliver brief conversations in the form of integrative mental health support, psychoeducation, and reminders. Tess serves as a therapeutic tool or resource that can be used as an adjunct to therapy that supports an integrative approach and is not intended to replace the role of a therapist.

Tess was inspired by the first conversational AI, ELIZA, which examined natural language communication between people and machines in the 1960s [19]. Where ELIZA was limited, Tess has excelled with the rapid advancement of technology and machine learning strategies that help improve AI memory and emotion identification.

The novelty of Tess is that it is a customizable platform, which allows for content to be tailored aligning with a specific form of treatment or user demographics, for example, 1 large health system in the United States customized Tess to deliver interventions based on motivational interviewing and behavioral activation to help reinforce weight management goals in a group of adolescents suffering from prediabetes symptoms (unpublished data [20]). To prevent misuse of the system, organizations are educated on Tess’ ethical AI code, which includes principles from the 2018 Lords Report [21] and the American Psychological Association Code of Conduct [22].

##### Technical Overview

Tess is maintained using a combination of technologies, emotion algorithms, and machine learning techniques to support a variety of features. Collaboration with mental health and emotion experts is a critical element of Tess’ capacity for success. All content is developed, screened, and matched to specific user inputs (ie, emotions and topics) by experienced professionals. The partnership required between humans and technology to create and maintain the chatbot is made possible through an administration panel that may be integrated with existing electronic health record systems. Each organization or clinician is provided a log-in with restricted access to manage their clients (Tess users) and content. For this study, the principal investigator and authors were provided with unique log-ins to a restricted view of participant interactions with Tess. Personally identifiable information was removed from all transcripts. Processing and storage are done on secure servers that meet Health Insurance Portability and Accountability Act–compliant regulations. Data are stored within the country of residence for all participants given access.

Tess can be configured to deliver services through existing communication channels such as Facebook messenger, Slack, and short messaging service text messaging, without requiring users to download an app. Users can access Tess using a mobile phone number or through their personal accounts associated with a specific communication channel. Tess is capable of interpreting free text messages; alternatively, users can opt for preselected responses similar to existing chatbots. This enhances Tess’ capacity to deliver more personalized and integrative interventions.

##### Customization

Content was specially selected, expanded upon, and tested by mental health professionals for the purpose of this study. Furthermore, 30 min to 1-hour interviews were conducted with students, professors, and university counselors to support content development. User acceptance testing was conducted with a small group of students, which provided feedback to enhance the quality and reliability of interventions and scripts. A modest amount of emojis were included in the conversations to increase user engagement.

To support the evaluation of duration and frequency of automatic messages from Tess, the test groups were assigned to 1 of the 2 experiments. Group 1 received daily messages from Tess over a 2-week period, introducing new topics or following up on previously discussed concerns. Group 2 received the same content and option for follow-up messages from Tess, but with biweekly messages over a 4-week period. It is of note that Tess was disguised as *Zara* for this study to prevent bias in the unlikely chance that participants had been exposed to Tess through another initiative.

Although Tess is capable of connecting users with a counselor in case of crisis, this study limited crisis support to match the methodology of a previous study [10]. If users reported suicidal or homicidal ideation or indicated a crisis, Tess provided numbers to the national suicide prevention hotline, crisis text line, and 911 and encouraged the user to end the chat and reach out for professional help.

##### Integrative Support Approach

Tess delivers mental health interventions that have repeatedly been shown to reduce symptoms of depression and anxiety, such as CBT [23], which maintains a strong evidence base [24]. In addition to CBT, Tess can also deliver a variety of similar, clinically proven therapies, dependent on both the emotions reported by the individual and the nature of their concern. These include interventions based on the transtheoretical model [25], emotionally focused therapy [26,27], solution-focused brief therapy [28], motivational interviewing [29], and more. By interacting with Tess, users experience the benefits of journaling, which has been shown to increase the positive perception of experiences [30] and significantly improve self-efficacy [31].

During the study, Tess delivered interventions rooted in a variety of psychological modalities such as CBT, mindfulness-based therapy, emotionally focused therapy, acceptance and commitment therapy, motivational interviewing, self-compassion therapy, and interpersonal psychotherapy. For example, journaling and relaxation strategies are used across multiple modalities, although the strategy and language used to deliver these interventions vary. Tess is structured to reply with prescripted statements, reviewed by mental health professionals, to replicate an empathic response that is appropriate to the participants’ inputted emotion or concern [32]. Specific interventions are delivered based on the users’ reported mood. For example, a participant indicating that he or she feels anxious may be offered a relaxation strategy to cope in the moment.

Just as therapists adjust their style to accommodate a client’s therapeutic preference over time, Tess gathers feedback to deliver interventions that best meet a user’s needs. After every intervention, Tess asks a simple question such as “was that helpful?,” to which user replies are coded as either positive, negative, or neutral. For example, if a user responds positively (ie, yes, thank you) to a CBT-based intervention and negatively (ie, no, not really) to self-compassion therapy, Tess will deliver more interventions rooted in CBT. For users who respond negatively or neutrally, Tess will continue to offer alternative interventions until the user responds positively or voluntarily ends the conversation.

#### Information Control Condition

Participants in the information-only control group were provided with an electronic link to the NIMH’s eBook on depression among college students [33]. The eBook is free for the public and provides evidence-based information and resources to help students identify, and seek treatment for, symptoms of depression. It also recommends additional literature and provides helpline numbers. Although participants in this group were not granted access to Tess during the course of the study, they were provided access on completion as an additional source of support.

### Measures

Participants were invited to take the following assessments via a closed email survey so that only those who were invited could gain access. All assessments were delivered through Google Forms.

#### Depression

Depressive symptom severity was assessed using the PHQ-9 [34]. The PHQ-9 is a self-report instrument with nine items and asks participants about depressive symptoms experienced in the last two weeks. Participants rate the frequency of each symptom utilizing a four-point scale ranging from 0 (not at all) to 3 (symptom experienced nearly every day). Therefore, total scores can range from 0 to 27, with higher scores indicating greater severity of depressive symptoms. Established guidelines for the PHQ-9 indicate that scores of 5 represent the threshold for mild depressive symptoms, 10 for moderate symptoms, 15 for moderately severe symptoms, and 20 for severe symptoms.

#### Anxiety

Anxiety symptom severity was assessed using the 7-item Generalized Anxiety Disorder (GAD-7) [35]. The GAD-7 is a self-report measure with seven items. Similar to the PHQ-9, the GAD-7 evaluates anxiety symptoms experienced during the previous two weeks. It utilizes a comparable four-point scale, ranging from 0 (no anxiety symptoms) to 3 (experiencing anxiety symptoms nearly every day), with total scores ranging from 0 to 21. Severity cutoff scores for the GAD-7 are 5 (mild anxiety), 10 (moderate), and 15 (severe).

#### Positive and Negative Affect Schedule

The Positive and Negative Affect Schedule (PANAS) [36] is a 20-item self-report measure of current positive and negative affect. Half of the items represent positive affect (ie, interested, excited, and determined), whereas the other half are indicative of negative affect (ie, hostile, scared, and ashamed). Items are scored on a 1 (very slightly or not at all) to 5 (extremely) scale, with higher scores representing higher affect. Scores range from 10 to 50, and positive and negative affect are summed independent of each other.

#### User Satisfaction and Engagement

A user satisfaction survey was created, tested for usability and technical functionality, and delivered to all participants at the end of the study to gather qualitative results. The survey included 9 questions, with 4 scaled questions, such as: “How satisfied were you overall?” and “How satisfied were you with the content?” as well as 2 open-ended questions, such as “What was the best thing about using the chatbot?” The remaining 3 questions were forced choice with response options of yes or no, such as “Did you learn anything new?.” Finally, for test group participants only, we measured engagement based on the number of messages exchanged between Tess and the participants per group and in total.

#### Statistical Analysis

Analyses were conducted using SPSS. A multivariate analysis of covariance (MANOVA) was used to compare the anxiety (GAD-7), depression (PHQ-9), and PANAS scale means of male and female students for the 3 groups, namely, control, group 1 (Tess for 2 weeks), and group 2 (Tess for 4 weeks). The multivariate analysis showed significance between the control group and group 1, *F*_3_=3.146, *P*=.3. The univariate *F* tests for anxiety and depression were significant across all scales, between control and group 1, *F*_2_=3.491, *P*=.3 for PANAS, *F*_2_=4.037, *P*=.02 for PHQ, and *F*_2_=4.497, *P*=.01 for GAD-7. Thus, anxiety and depression were significantly decreased through students’ use of Tess.

A post hoc analysis showed a significant difference was found with the Tukey’s Honestly Significant Difference Test (Multimedia Appendix 4) on the PANAS between the control and group 1. A statistically significant difference was found on the PHQ-9 between the control and group 1 at an alpha level of .05. For multivariate tests, the tests of between-subject effects, and intervention sample GIF, see Multimedia Appendix 5,Multimedia Appendix 6, and Multimedia Appendix 7, respectively. This finding confirms Tess was helpful in decreasing depressive symptoms. A statistically significant difference was found on the GAD-7 between the control group and both groups 1 and 2, at an alpha level of .05. This finding supports the hypothesis that Tess would be helpful in decreasing anxiety symptoms. Due to attrition, 1 control group participant was dropped out and not included in the total N for the control group.

## Results

### Participant Demographics

Table 1 shows the demographic information and baseline scores on clinical variables for those with data from the entire sample (N=74). Participants were aged on an average 22.9 years and over two-thirds were female. The majority of participants were Asian (37/74, 51%) and white (32/74, 41%).

In the control group, 67% (16/24) of participants were females, 29% (7/24) were males, and 4% (1/24) identified as nonconforming. The average age for the control group was 22.5 years. The majority of the control group participants were white (11/24, 46%). The remainder of the control group participants were Asian (8/24, 33%), other (3/24, 13%), and African American (2/24, 8%).

Group 1 consisted of 17 (17/24, 71%) females and 7 (7/24, 29%) males out of 24 participants. The average age for group 1 was 24.1 years. This group consisted of mostly white (13/24, 54%) and Asian (11/24, 46%) participants.

Group 2 consisted of 19 (19/26, 73%) females and 7 (7/26, 27%) males out of 26 participants. The average age for group 2 was 22.2 years. This group had mostly Asian participants (18/26, 69%), with 31% (8/26) being white.

### Participants’ Clinical Variables

Table 1 shows the scores for the 2 scales and for the subscales of the PANAS. In the control group, the average PHQ-9 score was 8.17, for group 1 it was 6.67, and for group 2 it was 7.04. The GAD-7 had an average score of 9.46 for the control group, 6.71 for group 1, and 7.5 for group 2. The average positive affect scale of the PANAS for the control group was 22.13, for group 1 it was 19.88, and for group 2 it was 21.31. The average negative affect scale of the PANAS for the control group was 15.75, for group 1 it was 13.08, and for group 2 it was 14.38.

**Table 1:** Demographic and clinical variables of participants at baseline.

Demographic and clinical variables		Information control	Tess group 1	Tess group 2
**Scale, mean (SD)**				
	Depression (PHQ^a^-9)	8.17 (4.2)	6.67 (4.6)	7.04 (4.9)
	Anxiety (GAD^b^-7)	9.46 (3.9)	6.71 (4.0)	7.5 (4.9)
	Positive affect	22.13 (1.4)	19.88 (1.4.)	21.31 (1.3)
	Negative affect	15.75 (1.3)	13.08 (1.3)	14.38 (1.3)
Age in years, mean (SD)		22.5 (4.0)	24.1 (5.4)	22.19 (2.8)
**Gender, n (%)**				
	Female	16 (67)	17 (71)	19 (73)
	Male	7 (29)	7 (29)	7 (27)
	Nonconforming	1 (4)	0 (0)	0 (0)
**Ethnicity, n (%)**				
	African American	2 (8)	0 (0)	0 (0)
	Asian	8 (33)	11 (46)	18 (69)
	White	11 (46)	13 (54)	8 (31)
	Other	3 (13)	0 (0)	0 (0)

^a^PHQ: Patient Health Questionnaire.

^b^GAD: Generalized Anxiety Disorder Scale.

### Analysis

Statistical power calculations using MANOVA revealed a moderate to large effect size (Cohen *d*=0.68) for depression, with an alpha at 5%.

#### The Patient Health Questionnaire-9

**Figure 2:**
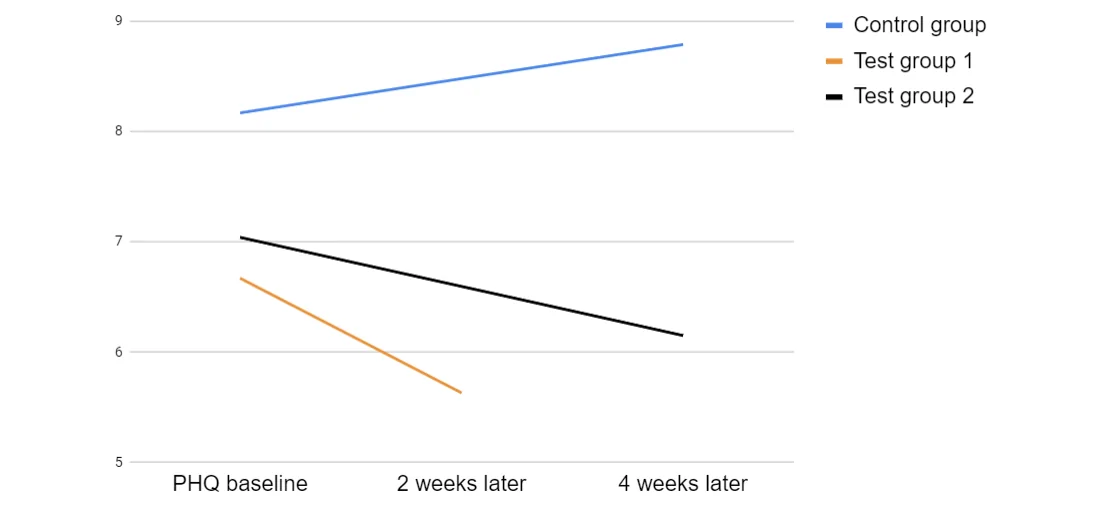
Change in depression by group (patient health questionnaire-9 score).

A statistically significant difference was found between the control group and group 1, which had unlimited access to Tess with daily check-ins for 2 weeks (*P*=.02) as measured by the PHQ-9. Figure 2 shows that MANOVA revealed a significant group difference on depression such that for the test group participants, symptoms of depression over the study period were significantly reduced, whereas for the information control group, their symptoms of depression were increased. Although the increase found in the control group is probably because of the inability to adjust for potential confounding variables, participants in this group may have experienced a slight increase in symptoms over time. Overall, these findings suggest that Tess was helpful in decreasing depressive symptoms.

#### Generalized Anxiety Disorder-7

A statistically significant difference was found between the control group and both test groups 1 and 2 for symptoms of anxiety as measured by the GAD-7. Group 1 (*P*=.045) and group 2 (*P*=.02) reported a significant reduction in symptoms of anxiety, whereas the control group did not.

**Figure 3:**
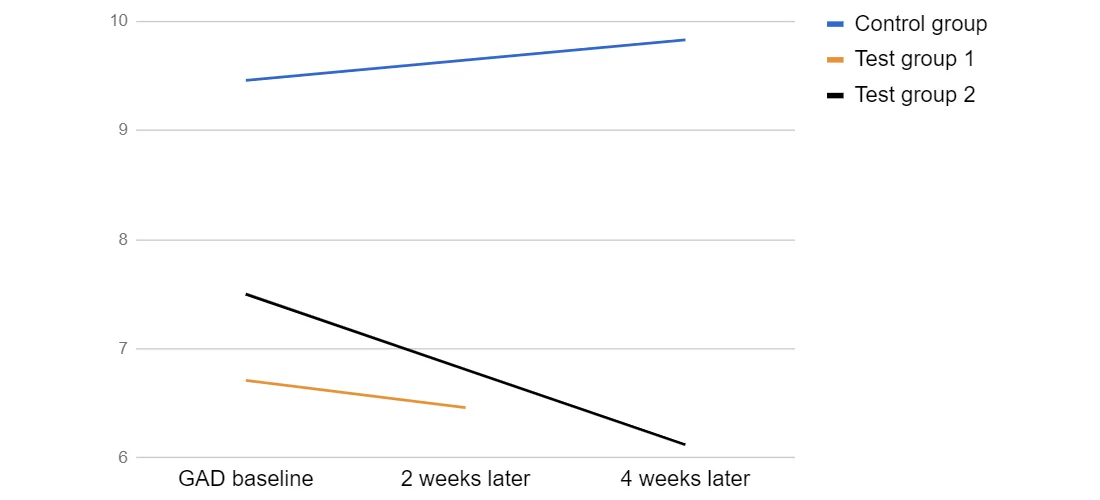
Change in anxiety by group (generalized anxiety disorder-7 score).

A statistically significant difference was found on the GAD-7 between the control group and both groups 1 and 2. For group 1, the *P* value of .045 was statistically significant at an alpha level of .05. For group 2, the *P* value of .02 was statistically significant at an alpha level of .05. Figure 3 shows that MANOVA revealed a significant group difference on anxiety such that those in the test group experienced significantly reduced symptoms of anxiety over the study period as measured by the GAD-7, whereas those in the information control group experienced increased symptoms of anxiety. This increase might be explained similarly to the aforementioned depressive scores—inability to adjust for potential confounding variables, or simply that, participants may have experienced a slight increase in symptoms over time. These findings support the hypothesis that Tess would be helpful in decreasing anxiety symptoms.

#### Positive and Negative Affect Schedule

A statistically significant difference was found on the PANAS between the control group and group 1 (*P*=.03) and suggests that Tess did impact the scores.

#### User Satisfaction and Engagement

Table 2 shows the difference between the information control group and the test group based on answers for the user satisfaction survey to gather qualitative results. This table shows a significant difference between both groups. For example, 86% (43/50) participants were overall satisfied with Tess and only 60% (14/24) with the eBook. Moreover, 80% (40/50) learned something new from Tess, and 43% (10/24) learned something new from the eBook.

**Figure 4:**
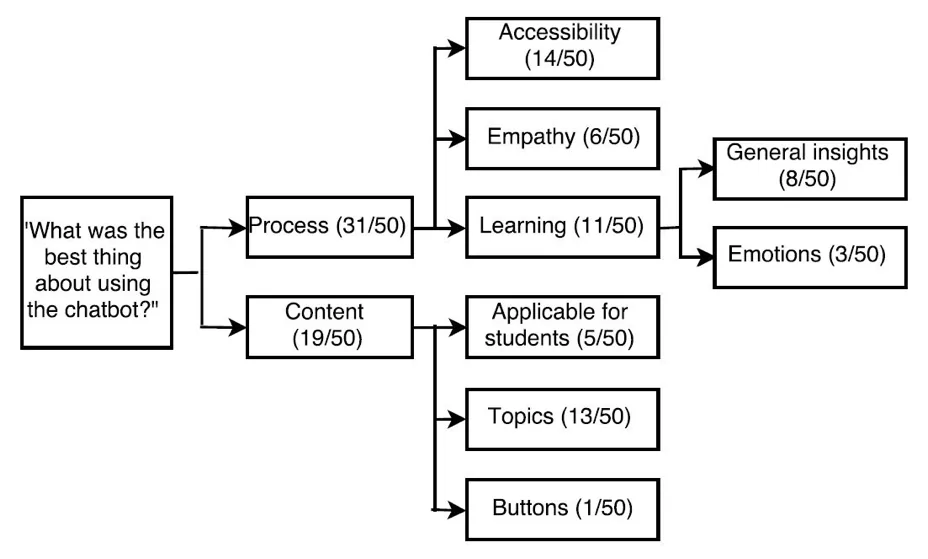
Thematic flow of participants’ most favored features while interacting with Tess.

Figure 4 shows a thematic map of participants’ responses to the question “What was the best thing about your experience using Tess?” Two major themes emerged in respect to this question: process and content. In the process theme, the subthemes that emerged were accountability from accessibility (noted by 15/50 participants); the empathy that the bot showed (6/50 participants); and the learning that the bot facilitated (11/50 participants), which in turn was divided into further subthemes of emotions and general insights.

**Table 2:** Qualitative results to post survey questions.

Post survey questions	Control group (N=24), n (%)	Tess group (N=50), n (%)
Overall satisfaction	14 (60)	43 (86)
Content satisfaction	15 (65)	40 (80)
Extend emotional awareness	17 (73)	43 (86)
Learned something new	10 (43)	40 (80)
Information relevant to everyday life	11 (47)	40 (80)
More comfortable with therapeutic process	11 (47)	32 (64)

**Figure 5:**
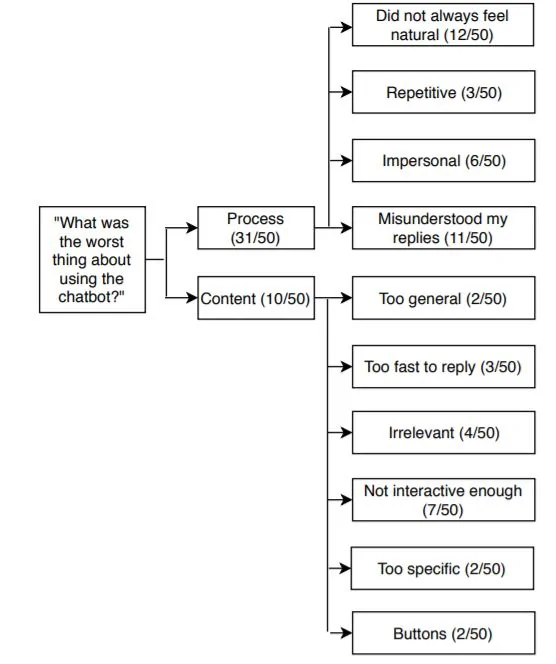
Thematic flow of participants’ least favored features while interacting with Tess.

Figure 5 illustrates a thematic map of participants’ responses to the question, “What was the worst thing about your experience with Tess?” Two themes emerged: process (31/50 participants) and problems with content (19/50). The most common subtheme to emerge among the process violations related to the limitations in natural conversation (12/50) and the bot not being able to understand some responses or getting confused when unexpected answers were provided by participants (11/50). Problems with content were described by 20% (10/50) of participants, most of which related to not enough interactivity (7/50, 14%).

A total of 48 open comments were received as feedback from 50 participants. Overall, 2 participants appeared to find their interaction with Tess to be particularly meaningful:

Based on our interactions I do somewhat feel like I’m talking to a real person and I do enjoy the tips you’ve given. In that sense, you’re better than my therapist in that she doesn’t necessarily provide specific ways I can better myself and problems.

I’ve been learning new things and I have some ideas on ways I can make small changes that could help me!.

Engagement was measured based on the number of messages exchanged between Tess and the participants. The X2AI Inc administration panel was used to calculate engagement metrics reported in this section. Participants from both test groups exchanged a total of 14,238 messages with Tess. Group 1 exchanged an average of 283 messages with daily pings and unlimited access to Tess for 2 weeks (SD 147.6; median 278; range 72-755). Group 2 exchanged an average of 286 messages with biweekly pings and unlimited access to Tess for 4 weeks (SD 104.6; median 288; range 133-535). Although we were unable to measure engagement of the NIMH eBook as it was not possible to track page views or URL click rates, 15 (15/24, 63%) control group participants reported that they were satisfied with the content supplied.

## Discussion

### Principal Findings

The objective of this study was to assess the feasibility and efficacy of using an integrative psychological AI to reduce self-identified symptoms of depression and anxiety in college students. The methodology and results aligned with those from a previous randomized trial, which examined the potential for a chatbot to deliver CBT-based interventions to college students [10]. Our hypotheses were that students would experience a greater reduction in symptoms of (1) depression and (2) anxiety after interacting with the integrative psychological AI for a period of 2 or 4 weeks when compared with participants in the information-only control group. Furthermore, we predicted that the test groups would report a more engaging and convenient experience than participants from the control group.

Results revealed that both test groups 1 and 2 experienced a significant reduction in symptoms of anxiety with unlimited access to Tess over the course of 2 or 4 weeks. Furthermore, the test group that received daily check-ins from Tess over 2 weeks experienced a significant reduction in symptoms of depression. Participants who interacted with Tess displayed higher levels of engagement and overall satisfaction than those from the control group. Test group participants indicated that the content was more relevant to their everyday life and made them more comfortable with the therapeutic experience.

### Limitations

Although this study included participants across 15 universities across the United States, the generalizability of results is limited, particularly as socioeconomic status was not formally assessed. The recruitment method further limits generalizability as we are unable to evaluate differences between the participants in this study who were recruited via social media and individuals who may use Tess but are not active on social media. The methodology called for 2 test groups and 1 control group, making the number of participants per group more limited. In addition, this study did not collect follow-up data to assess if benefits were sustained over time. Alternative to previous studies, the control group experienced a slight increase in symptoms of anxiety and depression, suggesting that the eBook was not a sufficient form of mental health support. One possible explanation for this outcome is that the eBook may have increased awareness of symptoms without providing ongoing treatment, leading to an increase over time. One study revealed that consumers of self-help books are more sensitive to stress and show higher depressive symptomatology [37]. Due to recruiting participants from a nonclinical sample, baseline depression (measured by PHQ-9) and anxiety (measured by GAD-7) scores were low. Therefore, additional research needs to be done to assess the feasibility of Tess in supporting individuals with clinical levels of depression and anxiety. Future studies should include control conditions that allow for a more direct comparison between delivery of services such as traditional therapy, as well as tech-based solutions, including teletherapy, interactive Web-based courses, and virtual reality.

Traditional therapeutic methods allow for emotional assessment on many different levels, including facial expressions, body cues, tone of voice, and language. The psychological AI used in this study delivered interventions via conversation, and therefore emotion identification was limited to language. It is unclear how much this limited the psychological AI’s assessment of emotion, as language is the most readily available nonphenomenal access people have to emotions. Assessing emotion through facial expressions [38,39] appears unreliable because of the overlap of expressive characteristics among seemingly basic emotions, resulting in the taxonomy of facial expressions not adequately describing the taxonomy of emotions [15].

Finally, the system errors, as expressed during qualitative feedback, are explained by the research team’s limited resources and attempt to keep all content approved by experts intact. During the study, changes to the system were restricted, and so the research team was unable to report errors related to natural language processing or emotion mismatch until the completion of the study.

### Comparison With Prior Work

Aligned with results from a previous study, using Tess was associated with a significant reduction in depression and anxiety as measured by the PHQ-9 and GAD-7, respectively. The effect size (Cohen *d*=0.68) for depression was moderate and greater than previously published studies [10,40-42] that measured the efficacy of using alternative mobile app interventions to relieve symptoms of depression. This study included 2 test groups to evaluate differences in symptom reduction based on 2- to 4-week intervention periods. The effect size for reduction of symptoms is aligned with that found by a previous study when delivering a CBT-based chatbot to college students in the United States [10]. Although speculative, the greater effect size found in this study may be due, in part, to the integrative mental health approach applied to deliver more personalized interventions. In addition, the content used to create Tess conversations was derived from written transcripts that allowed participants to respond with free text, versus predominantly using buttons to receive videos and other resources on a timely basis as the chatbot in a previous study did [10].

With a growing demand for scalable solutions that deliver more cost-effective mental health support, it has been shown that CAT is capable of delivering empirically validated treatments for depression and anxiety [26]. Preliminary studies suggest that self-help computer-based cognitive and behavioral interventions produce similar outcomes to in-person treatment [27]. Clinical treatment outcomes have been higher for patients prescribed to use psychotherapeutic computer programs compared with programs that are delivered in a self-help format with no clinician involvement [38].

Previous studies suggest that individuals are more willing to disclose personal information to a psychological AI than to a *virtual therapist* purportedly operated by a human [9,10,43]. This was supported by feedback given by student participants who engaged on a more personal level with Tess for this study. Students reported:

I do somewhat feel like I’m talking to a real person.

...you’re better than my therapist [who] doesn’t necessarily provide specific ways I can better myself.”

And that Tess was able to:

coach [the participant] through a difficulty.

These comments reinforce the potential for psychological AI to remove barriers and stigma and operate as an adjunct to traditional therapeutic methods.

### Conclusions

This study reveals that AI offers a cost-effective and accessible mental health solution and may serve as a scalable tool to complement traditional treatment methods. Although integrative psychological AI is not designed or intended to replace the role of a trained therapist, Tess emerges as a feasible option for delivering emotional support. The results support and expand on findings from a previous randomized controlled trial [10] and demonstrate that psychological AI has the potential to reduce symptoms of depression and anxiety by delivering CBT-based interventions in the form of conversations.

## Appendix

Multimedia Appendix 1 Recruitment flyer.

Multimedia Appendix 2 Consolidated Standards of Reporting Trials-eHealth checklist.

Multimedia Appendix 3 Informed consent.

Multimedia Appendix 4 Multiple comparisons table.

Multimedia Appendix 5 Multivariate test table.

Multimedia Appendix 6 Tests of between-subjects effects.

Multimedia Appendix 7 Intervention sample GIF.

## Abbreviations

AI: artificial intelligence
CAT: computer-assisted therapy
CBT: cognitive-behavioral therapy
DSM: Diagnostic and Statistical Manual of Mental Disorders
GAD: generalized anxiety disorder
MANOVA: multivariate analysis of covariance
mHealth: mobile health
NIMH: National Institute of Mental Health
PANAS: positive and negative affect scale
PHQ: Patient Health Questionnaire
