# Correction: Using Psychological Artificial Intelligence (Tess) to Relieve Symptoms of Depression and Anxiety: Randomized Controlled Trial

**DOI:** 10.2196/108162

**Published:** 2026-08-26

**Authors:** Russell Fulmer, Angela Joerin, Breanna Gentile, Lysanne Lakerink, Michiel Rauws

**Affiliations:** 1 School of Medicine Northwestern University Evanston, IL United States; 2 X2 AI Inc San Francisco, CA United States; 3 Saxion University of Applied Sciences Enschede The Netherlands

In “Using Psychological Artificial Intelligence (Tess) to Relieve Symptoms of Depression and Anxiety: Randomized Controlled Trial” [1], the following corrections were made in response to a request by Dr Matthew Swift and the JMIR editorial team. Each correction is listed below.

An empathetic response issued by Tess was indicated by authors RF and AJ to be an oversight in the originally published article.

The following sentence has been deleted from the manuscript:

For example, in response to endorsed loneliness, Tess replied “I’m so sorry you’re feeling lonely. I guess we all feel a little lonely sometimes,” or Tess showed excitement by replying, “Yay, always good to hear that!.”

Our rationale for removal, rather than correction, is rooted in current data availability. During the study, X2AI Inc. aligned its data retention policies with California state guidelines, requiring clinical data to be stored for 7 years. Because we are now beyond that 7-year threshold and X2AI Inc. is no longer in business, the original chat transcripts have been expunged. Without access to the primary data to definitively verify a replacement quote, we believe removing the sentence entirely is the most scientifically responsible approach to address this oversight.

A statement within the Methods that more clearly indicates that the 2018 study was designed and executed to mirror the 2017 study by Fitzpatrick et al has been added:

The methodology of our study was developed to closely parallel that of Fitzpatrick et al [10] to further contribute to the emergence of AI as a supportive mental health tool. A major goal of our research was to compare findings across conversational agents, in this case, Tess and Woebot. By aligning our research design, participant procedures, and primary outcome measures with the Fitzpatrick study, we intended to assess whether an alternative AI chatbot, Tess, could also yield positive outcomes and effectively reduce symptoms of depression and anxiety. This parallel approach was designed to bolster the broader literature about conversational agents as therapeutic tools. The primary methodological difference was the use of a different AI chatbot rather than the platform evaluated by Fitzpatrick et al [10].

The description of measures within the Methods section has been reworded to differ from that of the Fitzpatrick study. While descriptions of the 9-item Patient Health Questionnaire and 7-item Generalized Anxiety Disorder will be similar across published manuscripts, the JMIR editorial team has requested that these descriptions not be identical between articles.

The following subsection was revised from the following:

The Patient Health Questionnaire-9

The PHQ-9 [34] is a 9-item, self-report questionnaire that evaluates the frequency and severity of symptoms of depression within the previous 2 weeks. Each of the 9 items is based on the Diagnostic and Statistical Manual of Mental Disorders (DSM-4) criteria for major depressive disorder and can be scored on a 0 (not at all) to 3 (nearly every day) scale. The PHQ-9 is one of the most widely used, reliable, and validated measures of depressive symptoms. If a participant scores between 0 and 5, this indicates they do not experience symptoms of depression. Scores of 5 to 9, 10 to 14, 15 to 20, and >20 indicate mild, moderate, moderately severe, and severe depression, respectively.

This subsection now reads:

Depression

Depressive symptom severity was assessed using the PHQ-9 [34]. The PHQ-9 is a self-report instrument with nine items and asks participants about depressive symptoms experienced in the last two weeks. Participants rate the frequency of each symptom utilizing a four-point scale ranging from 0 (not at all) to 3 (symptom experienced nearly every day). Therefore, total scores can range from 0 to 27, with higher scores indicating greater severity of depressive symptoms. Established guidelines for the PHQ-9 indicate that scores of 5 represent the threshold for mild depressive symptoms, 10 for moderate symptoms, 15 for moderately severe symptoms, and 20 for severe symptoms.

Similarly, the following subsection was revised from the following:

Generalized Anxiety Disorder-7

The Generalized Anxiety Disorder 7-item scale (GAD-7) [35] is a valid, brief self-report tool to assess the frequency and severity of anxious thoughts and behaviors over the past 2 weeks. On the basis of the DSM-4 diagnostic criteria for GAD-7, the scores of all 7 items range from 0 (not at all) to 3 (nearly every day). If a participant scores less than 10, it indicates moderate anxiety. A score greater than 15 indicates severe anxiety.

This subsection now reads:

Anxiety

Anxiety symptom severity was assessed using the 7-item Generalized Anxiety Disorder (GAD-7) [35]. The GAD-7 is a self-report measure with seven items. Similar to the PHQ-9, the GAD-7 evaluates anxiety symptoms experienced during the previous two weeks. It utilizes a comparable four-point scale, ranging from 0 (no anxiety symptoms) to 3 (experiencing anxiety symptoms nearly every day), with total scores ranging from 0 to 21. Severity cutoff scores for the GAD-7 are 5 (mild anxiety), 10 (moderate), and 15 (severe).
