# Supplementary material for: Using Psychological Artificial Intelligence (Tess) to Relieve Symptoms of Depression and Anxiety: Randomized Controlled Trial
Source: JMIR Ment Health. 2018 Dec 13;5(4):e64. doi: 10.2196/mental.9782 (PMC6315222; doi:10.2196/mental.9782)
Supplement: Multimedia Appendix 1 [file mental_v5i4e64_app1.pdf]

CAN ARTIFICIAL INTELLIGENCE HELP ME COPE WITH DEPRESSION AND ANXIETY?

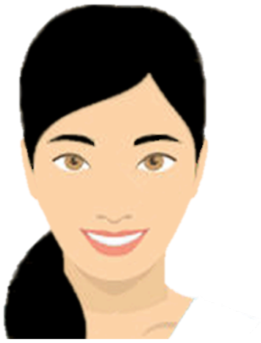

Zara is a psychological artificial intelligence chatbot that administers on-demand personalized psychotherapy, psychoeducation, and health-related reminders. Zara is equipped to talk about a wide range of student related subjects that are meant to decrease symptoms of anxiety and depression and help manage stress.

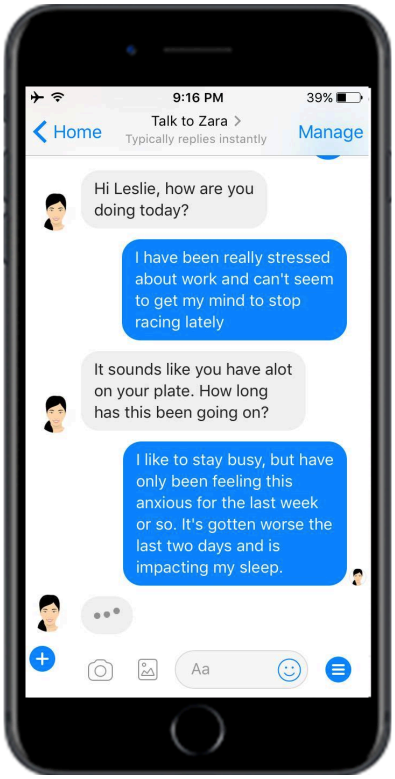

As a participant in this study, you will receive free mental health support, while helping to prove the efficacy of Zara in relieving symptoms of anxiety and depression. The study will begin **September 4, 2017** and end **October 11, 2017.**

To participate in this study you need to:

- Be 18+ years of age
- Be a student in the United States
- Have access to Facebook messenger

Please contact **Liz@x2ai.com** to receive more information.

Participants will receive $20 as a token of appreciation. We look forward to hearing from you!

THE NEW YORKER REUTERS The Washington Post
