# Supplementary material for: Using Psychological Artificial Intelligence (Tess) to Relieve Symptoms of Depression and Anxiety: Randomized Controlled Trial
Source: JMIR Ment Health. 2018 Dec 13;5(4):e64. doi: 10.2196/mental.9782 (PMC6315222; doi:10.2196/mental.9782)
Supplement: Multimedia Appendix 3 [file mental_v5i4e64_app3.pdf]

X2 Student Study | Using Artificial Intelligence to cope with Anxiety and Depression

Informed consent

INTRODUCTION

You are invited to join a research study to assess the efficacy of using artificial intelligence to reduce symptoms of depression and anxiety in students. Please take time to discuss the study with your family and friends, or anyone else as you wish. The decision to join, or not to join, is up to you.

Zara is a psychological artificial intelligence chatbot that administers on-demand personalized psychotherapy, psychoeducation, and health-related reminders. Zara is equipped to talk about a wide range of student related subjects that are meant to decrease symptoms of anxiety and depression and help manage stress.

As a participant in this study, you will receive free mental health support, while helping to prove the efficacy of Zara in relieving symptoms of anxiety and depression. This one month study will begin September 6, 2017 and end October 11, 2017.

To participate in this study you need to:
- Be 18+ years of age
- Be a student in the United States
- Have access to Facebook messenger

WHAT IS INVOLVED IN THE STUDY?

As a participant, you will be asked to spend 5-10 minutes taking a survey at the start and end of the study to help us understand your experience with anxiety and depression. Regardless of survey responses, participants will be divided in one of three groups. The first group will be asked to interact with Zara for 2 weeks every day, the second group will receive messages from Zara two times a week for 4 weeks and the third group will receive an online e-book about coping with depression and anxiety. All three groups will have the option to interact with our chatbot after the study for continued support.

The investigators may stop the study or remove participants at any time. You may withdraw or stop participating at any time.

BENEFITS TO TAKING PART IN THE STUDY?

It is reasonable to expect the following benefits from this research: free mental health support, while helping to prove the efficacy of Zara in relieving symptoms of anxiety and depression..

However, we can’t guarantee that you will personally experience benefits from participating in this study. Others may benefit in the future from the information we find in this study.

CONFIDENTIALITY

We will take the following steps to keep information about you confidential, and to protect it from unauthorized disclosure, tampering, or damage: Only the employees of the company X2AI will have access to the data and records of your conversations. Results will be reported in aggregate and no individual results will be revealed.

INCENTIVES

Participants will receive a $20 Amazon e-giftcard as a token of appreciation after completing the study. Completion includes signing the informed consent, completing both the baseline and post survey, and interacting with Zara throughout the study.

YOUR RIGHTS AS A RESEARCH PARTICIPANT?

Participation in this study is voluntary. You have the right not to participate at all or to leave the study at any time. Deciding not to participate or choosing to leave the study will not result in any penalty.

To withdraw early you can contact Lysanne Lakerink at **liz@x2ai.com**.

CONTACTS FOR QUESTIONS OR PROBLEMS?

Email Lysanne Lakerink at **liz@x2ai.com** if you have questions about the study, any problems, unexpected physical or psychological discomforts, any injuries, or think that something unusual or unexpected is happening.

Consent of Subject (or Legally Authorized Representative)

Signature of Subject or Representative Date

___________________________________ _________________

Upon signing, the subject or the legally authorized representative will receive a copy of this form, and the original will be held in the subject’s research record. Unless otherwise required by the HSRO, Exempt research does not require a signature. For all other research, in some cases it may be in the best interest of the subject not to collect a signature and the HSRO will advise you if that is the situation.
