# Supplementary figures and images for: Using Psychological Artificial Intelligence (Tess) to Relieve Symptoms of Depression and Anxiety: Randomized Controlled Trial
Source: JMIR Ment Health. 2018 Dec 13;5(4):e64. doi: 10.2196/mental.9782 (PMC6315222; doi:10.2196/mental.9782)

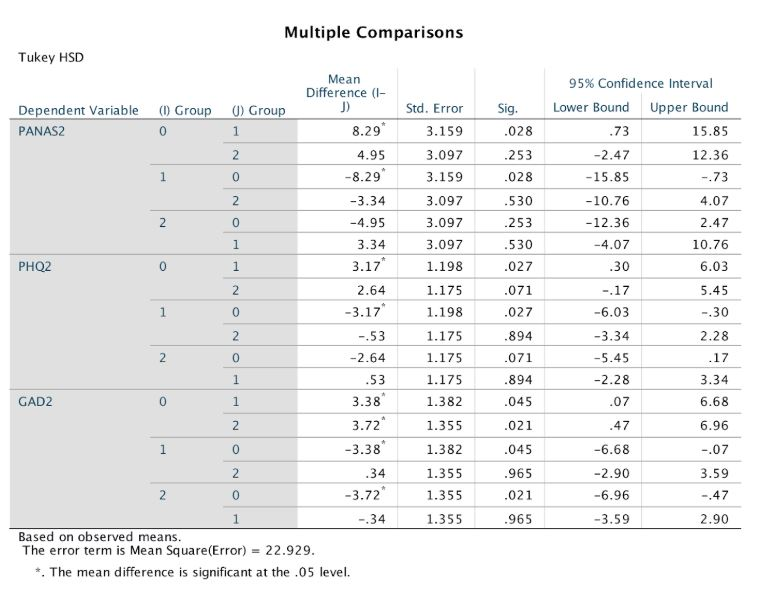

Supplement: Multimedia Appendix 4 [file mental_v5i4e64_app4.png]

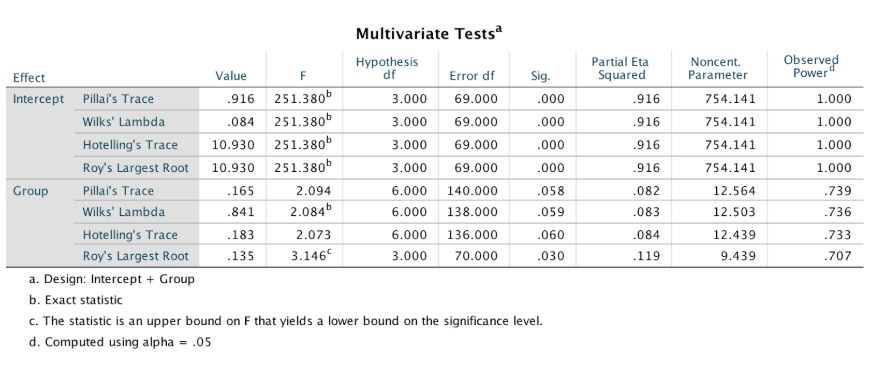

Supplement: Multimedia Appendix 5 [file mental_v5i4e64_app5.png]

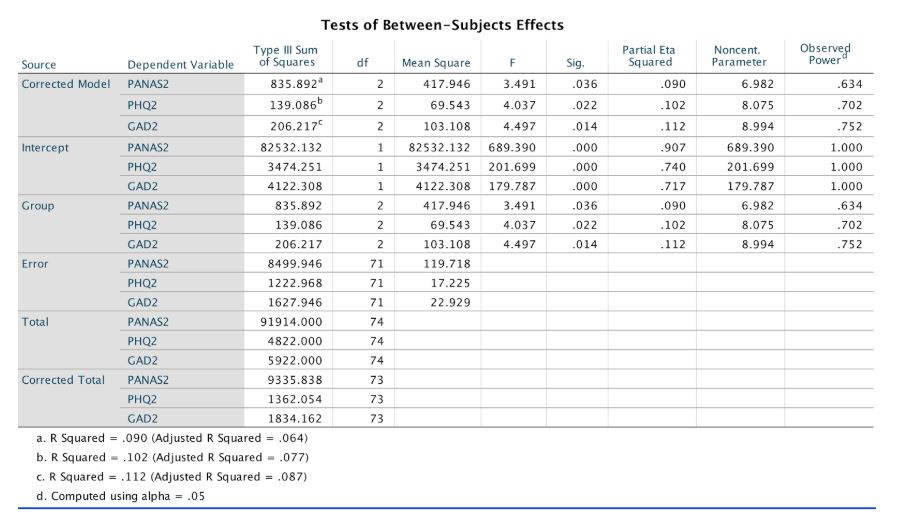

Supplement: Multimedia Appendix 6 [file mental_v5i4e64_app6.png]
